# Beyond Meatless, the Health Effects of Vegan Diets: Findings from the Adventist Cohorts

**DOI:** 10.3390/nu6062131

**Published:** 2014-05-27

**Authors:** Lap Tai Le, Joan Sabaté

**Affiliations:** Department of Nutrition, School of Public Health, Loma Linda University, CA 92350, USA; E-Mail: ltle@llu.edu

**Keywords:** vegetarian, vegan, lacto-ovo-vegetarian, Adventist, Health, AMS, AHS, cardiovascular, cancer, mortality

## Abstract

Vegetarians, those who avoid meat, and vegans, additionally avoiding dairy and eggs, represent 5% and 2%, respectively, of the US population. The aim of this review is to assess the effects of vegetarian diets, particularly strict vegetarian diets (*i.e.*, vegans) on health and disease outcomes. We summarized available evidence from three prospective cohorts of Adventists in North America: Adventist Mortality Study, Adventist Health Study, and Adventist Health Study-2. Non-vegetarian diets were compared to vegetarian dietary patterns (*i.e.*, vegan and lacto-ovo-vegetarian) on selected health outcomes. Vegetarian diets confer protection against cardiovascular diseases, cardiometabolic risk factors, some cancers and total mortality. Compared to lacto-ovo-vegetarian diets, vegan diets seem to offer additional protection for obesity, hypertension, type-2 diabetes, and cardiovascular mortality. Males experience greater health benefits than females. Limited prospective data is available on vegetarian diets and body weight change. Large randomized intervention trials on the effects of vegetarian diet patterns on neurological and cognitive functions, obesity, diabetes, and other cardiovascular outcomes are warranted to make meaningful recommendations.

## 1. Introduction

A recent Gallup Poll conducted in July 2012 reported that 5% of the American adult population is vegetarian. Only 2% reported adopting a vegan diet, in that they abstain from meat, fish, dairy, and eggs [1]. The attitudes towards vegetarianism have gained better acceptance; however, the percentage of Americans who consider themselves to be vegetarians is largely unchanged since 2001. For many individuals, the choice for adopting a vegetarian diet stems from various reasons including religious and ethical considerations [2], environmental impacts [3], and health benefits of a plant-based diet [4,5]. About three decades ago, the position statement of American Dietetic Association showed reasonable doubts about the nutritional adequacy and benefits of vegetarian diets [6]. However, the committee has changed its positions on vegetarian dietary recommendations [4,7]. This is the results of much research on vegetarian diets in the past 30 years. The increased number of publications on vegetarian nutrition and major contributions in nutritional epidemiology have contributed to better understanding of the connections between diet and disease [8]. In particular, studies of Adventists have provided compelling evidence that well-balanced vegetarian diets are nutritionally adequate, and associated with lower risk of chronic diseases [4].

Probably, the first thoughtful investigation on vegetarian diets was conducted by Mervyn G. Hardinge as a doctoral dissertation at Harvard in the early 1950s [9,10,11]. His research was initially met with resistance [12], but later became the founding Dean of Loma Linda University School of Public Health. His novel thinking opened up new hypotheses for subsequent research into vegetarian diets. Notably, the prospective epidemiological studies of Adventists, many of whom are vegetarians—vegans, lacto-ovo-vegetarians, semi-vegetarians, pesco-vegetarians; and about half adheres to omnivorous diets similarly to that of the general population [13]. Members of the Seventh-day Adventist religious denomination exhibits a variety of dietary habits, with a strong emphasis on health. Church doctrines recommend vegetarian practices and abstinence from the use of tobacco and alcohol. The range of diets consumed by Adventists is broad and distinct from the typical Western diets. Thus, this presents an ideal opportunity to compare various vegetarian dietary patterns, while controlling for known non-dietary confounders, such as the use of alcohol and tobacco [14].

The aim of this review is to assess the health and disease outcomes of vegetarian diets with regards to chronic diseases. Are the health effects of those who adhere to strict vegetarian (*i.e.*, vegan) and lacto-ovo-vegetarian diets differ from non-vegetarians? Thus, the purpose of this review is to summarize available evidence from the Adventist cohorts of potential health effects for vegan and lacto-ovo-vegetarian diets on disease and mortality; and to discuss the limitations of these diets in order to provide practical dietary recommendations.

## 2. Methods

In order to identify relevant articles, we conducted an independent literature review using PubMed/Medline and EMBASE databases through 28 January, 2014. The following search terms were used: “Adventist vegetarian health” (49 articles); “vegan Adventist health” (46 articles); “Adventist vegetarian cancer” (32 articles); “Adventist health BMI” (28 articles); “Adventist mortality vegetarian” (16 articles); “lacto-ovo-vegetarian Adventist” (11 articles); “Adventist health study dietary pattern” (8 articles); “Adventist vegetarian diabetes” (6 articles); and “dietary patterns Adventist mortality” (3 articles). Additionally, we cross-referenced the articles with the Adventist Health Study database of 348 articles. For the purpose of this study, only relevant articles were included using the following inclusion criteria: (1) large prospective cohort studies of Adventists; (2) reports with clearly defined dietary patterns as exposures; and (3) data of vegetarians compared to non-vegetarians (lacto-ovo-vegetarians and/or vegans) on cardiometabolic factors, cancer-related sites, and/or mortality. Exclusion criteria were: animal studies, case studies with small sample size, studies of Adventists outside of the US and Canada; studies reporting only nutrients, foods, and food groups as dietary exposures; and studies not reporting relevant health outcomes.

After careful review of thirteen articles relevant to the aim of this report, data were abstracted and tabulated according to pre-established criteria, related to the classification of the dietary patterns (Table 1), and characteristics of the study cohorts (Table 2). Eight articles were included in the comparisons between vegetarians and non-vegetarians for cardio-metabolic factors (Table 3), cancer-related sites (Table 4), and mortality (Table 5). Six articles were included in the review for lacto-ovo-vegetarians and vegans compared to non-vegetarians on cardiometabolic factors (Table 6), cancer-related sites (Table 7), and mortality (Table 8). The results extracted from these studies used various parameter estimates including means and standard deviation (SD), odd ratio (OR), relative risk (RR), hazard ratio (HR), and standard mortality ratio (SMR). Most studies reported results using the non-vegetarian group as the reference. In order to unify the results across all studies, reciprocal of the parameter estimates and confidence intervals were calculated with non-vegetarian group as the reference. In this report, we adhered to the commonly used definitions of vegetarian dietary patterns in the Adventist Health Study cohorts, as summarized in Table 1. Results are presented in a tabular format.

**Table 1:** Classification of dietary patterns *.

Dietary Pattern	Definition	Beef	Poultry/Fish	Dairy/Eggs
**Non-vegetarian**	Eat red meat, poultry, fish, milk, and eggs more than once a week	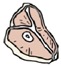	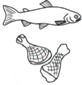	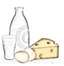
**Semi-vegetarian**	Eat red meat, poultry, and fish less than once per week and more than once per month	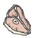	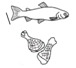	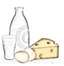
**Vegetarian**				
**Pesco-**	Eat fish, milk, and eggs but no red meat nor poultry	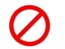	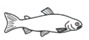	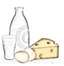
**Lacto-ovo-**	Eat eggs, milk, or both but no red meat, fish, nor poultry	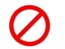	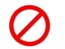	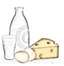
**Vegan**	Eat no red meat, fish, poultry, dairy, and eggs	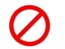	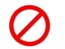	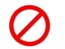

* Adapted from Gary E. Fraser [15].

Three separate prospective Adventist cohort studies, whose dietary practice and health outcomes of participants were followed. The first major Seventh-day Adventist prospective cohort study was called the California Adventist Mortality Study (AMS) [14,15,16]. This study was followed-up to disease mortality collected between 1960 and 1976, from 22,940 participants, of which 64.6% were females. The subsequent cohort study of Adventists was the Adventist Health Study-1 (AHS-1) [17], which started in 1976 to 1988 at the end of follow-up. It was designed to assess diet-disease associations and collect fatal and non-fatal incident events from 34,198 participants living in California, ages 25–90 years. The third and on-going prospective cohort study was the Adventist Health Study-2 (AHS-2) [14]. AHS-2 began in 2002 with the aim to evaluate the role of diets and lifestyle factors that may be involved in cancer-related outcomes. This is a cohort of more than 96,000 participants from US and Canada, ages 30–112 years [14]. Dietary data were measured at baseline using self-administered food frequency questionnaire to assess usual dietary intake, and types of dietary pattern were defined. Cancer cases were identified and matched with state tumor registries. Morality data were obtained from the National Death Index. Table 2 summarizes the basic characteristics of the Adventist cohorts by location, subjects (*n*), age range (years), years of follow-up (years), outcomes of interest, and study design methodology, as detailed by Butler *et al*. [14,15,18,19,20].

**Table 2:** Characteristics of Adventist Mortality Study (AMS), Adventist Health Study-1 (AHS-1), and Adventist Health Study-2 (AHS-2).

Sources	Location	Number of Subjects (*n*)	Age Range (years)	Years of Follow-Up (years)	Outcomes of Interest	Study Design
**Adventist Mortality Study (AMS) [16]**	California	22,940 64.6% Female	35–90	1960–1976	Disease Mortality	Prospective
**Adventist Health Study-1 (AHS-1) [17]**	California	34,198 60.1% Female	25–90	1976–1982 (incidence) 1976–1988 (mortality)	Disease incidence & mortality	Prospective
**Adventist Health Study-2 (AHS-2) [14]**	50 U.S. States & Canada	96,194 65.1% Female	30–112	2002–(ongoing)	Disease Incidence & morality	Prospective

Adventist Cohorts and Study Participants

## 3. Results

For more than 96,000 participants from AHS-2 cohort, 48% were non-vegetarians, 6% were semi-vegetarians, 10% were pesco-vegetarians, 28% were lacto-ovo-vegetarians, and 8% were vegans at baseline. Table 3 presents cardiometabolic risk factors comparing vegetarian and non-vegetarian Adventists. For BMI, vegetarians were approximately 2–4 points lower than non-vegetarians. After adjusted for relevant confounders, vegetarians had 55% lower odds of developing hypertension. The odds of developing type-2 diabetes was 25% to 49% lower for vegetarians compared to non-vegetarians in different cohorts. The odds of developing metabolic syndrome (MetS) for vegetarians were about half compared to non-vegetarians.

**Table 3:** Cardiometabolic factors among vegetarian and non-vegetarian Adventists.

Cardiometabolic Factor	Person at-Risk	No. of Events	Parameter Estimates	Non-Vegetarian	Vegetarian	Cohort(s) & References
				Mean or OR (95% CI)		
**BMI** ^[a]^^,^* *Men* *Women*	34,192	-	Mean, kg/m^2^	26.2 (26.1, 26.4) 25.9 (25.8, 26.0)	24.3 (24.1, 24.4) 23.7 (23.6, 23.9)	AHS-1 [21]
**BMI** ^[a]^^,^*	773	-	Mean, kg/m^2^	29.6 (29.0, 30.3)	25.9 (25.2, 26.6)	AHS-2 [22]
**Hypertension** ^[b]^^,^^‡^ *Men* *Women*	34,192	-	OR	1 [*referent*]	0.45 (0.40, 0.51) 0.45 (0.41, 0.49)	AHS-1 [21]
**Diabetes** ^[b]^^,^^‡^ *Men* *Women*	34,192	-	OR	1 [*referent*]	0.51 (0.40, 0.64) 0.52 (0.44, 0.64)	AHS-1 [21]
**Diabetes Mellitus** ^[c]^^,^^♠^	8401	543	OR	1 [*referent*]	0.75 (0.57, 0.97)	AMS & AHS-1 [23]
**Metabolic Syndrome** ^[d]^^,†^	773	-	OR	1 [*referent*]	0.44 (0.30, 0.64)	AHS-2 [22]

Non-vegetarian is used as the referent group for comparison. OR = odd ratio; ^[a]^ Adjusted for age and diet status, * *p* *=* 0.0001; ^[b]^ Adjusted for age. Significantly different from non-vegetarians. ^‡^ *p* *=* 0.0001; ^[c]^ Multivariate logistic regression model adjusted for age, sex, and BMI; ^♠^ *p* < 0.005; ^[d]^ Logistic regression analysis adjusted for sex, ethnicity, smoking, alcohol intake, physical activity, and dietary energy intake; ^†^ *p* < 0.001.

Table 4 presents results comparing risks of all-cancer and cancer-specific sites between vegetarian and non-vegetarian Adventists. Vegetarians experienced a modest, 8% risk reduction for overall-cancer. For cancer-specific sites, vegetarians had approximately half the risk of developing colon cancer. Also, vegetarians had 23% risk reduction for cancer of the gastrointestinal tract. Vegetarians experienced a 35% risk reduction for prostate cancer compared to non-vegetarians. Similarly, vegetarians tended to have lower risk for cancer of the respiratory tract and overall-cancer. No significant differences between the diet groups were found for other site-specific cancers including lung, breast, and uterine.

**Table 4:** All-cancer and cancer-specific sites among vegetarian and non-vegetarian Adventists.

Cancer Sites	Person at-Risk	No. of Events	Parameter Estimates	Non-vegetarian	Vegetarian	Cohort(s) & References
				RR or HR (95% CI)		
**Colon** ^[a]^^,^*	34,198	107	RR	1 [*referent*]	0.55 (0.35, 0.81)	AHS-1 [21]
**Colon** ^[b]^^,†^	34,198	166	RR	1 [*referent*]	0.39 (0.19, 0.83)	AHS-1 [24]
**Gastrointestinal Tract** ^[c]^^,^^♠^	69,120	495	HR	1 [*referent*]	0.77 (0.63, 0.93)	AHS-2 [20]
**Lung** ^[h]^	34,198	45	RR	1 [*referent*]	0.86 (0.42, 1.79)	AHS-1 [21]
**Respiratory Tract** ^[c]^	69,120	170	HR	1 [*referent*]	0.75 (0.54, 1.04)	AHS-2 [20]
**Urinary Tract** ^[c]^	69,120	194	HR	1 [*referent*]	1.21 (0.89, 1.65)	AHS-2 [20]
**All-male Cancer** ^[d]^	24,446	553	HR	1 [*referent*]	0.94 (0.42, 2.07)	
**Prostate** ^[a]^	34,198	127	RR	1 [*referent*]	0.65 (0.44, 0.95)	AHS-1 [21]
**All-female Cancer** ^[e]^	44,674	801	HR	1 [*referent*]	0.97 (0.84, 1.13)	AHS-2 [20]
**Breast** ^[a]^	34,198	128	RR	1 [*referent*]	0.80 (0.56, 1.15)	AHS-1 [21]
**Uterine** ^[a]^	34,198	1.16	RR	1 [*referent*]	0.85 (0.58, 1.23)	
**Overall-Cancer** ^[f]^^,^^‡^	69,120	2939	HR	1 [*referent*]	0.92 (0.85, 1.00)	AHS-2 [20]
*Males* ^[g]^	24,446	1235	HR	1 [*referent*]	0.92 (0.81, 1.03)	
*Females* ^[f]^	44,674	1704	HR	1 [*referent*]	0.93 (0.84, 1.03)	

All-female cancer includes female breast, vulva, vagina, cervix uteri, corpus uteri, endometrial, uterus, and ovary. Cancer of the gastrointestinal tract includes esophagus, stomach, small intestine, colon, liver and bile ducts, gallbladder, biliary tract, and pancreas. All-male cancer includes prostate, penis, and testis. Cancer of the respiratory tract and intra-thoracic organs include nasal cavity, middle ear, larynx, trachea, bronchus, lung, heart, mediastinum, and pleura. Cancer of the urinary tract includes renal pelvis, ureter, kidney, and bladder. RR = relative risk; HR = hazard ratio. ^[a]^ Adjusted for age and sex; * *p* *=* 0.0032; ^[b]^ Adjusted for age, sex, and parental history of colon cancer; and relative to vegetarians with legume intake of <1 time/week; ^†^ *p* *=* 0.03; ^[c]^ Multivariate HR model adjusted for age, family history of cancer, education, smoking, alcohol, and BMI; ^♠^ *p* *<* 0.05; ^[d**]**^ Multivariate HR model adjusted for age, family history of cancer, education, smoking, and alcohol. Applicable only for male-specific cancer; ^[e]^ Multivariate HR model adjusted by race, family history of cancer, education, smoking, alcohol, age at menarche, pregnancies, breastfeeding, oral contraceptives, hormone replacement therapy, menopause status, and BMI; ^[f]^ HR adjusted by race, family history of cancer, BMI, education, smoking, alcohol, age at menarche, pregnancies, breastfeeding, oral contraceptives, hormone replacement therapy, and menopause status; ^‡^ *p* *=* 0.05; ^[g]^ HR adjusted by race, family history of cancer, BMI, education, smoking, and alcohol; ^[h]^ Adjusted for age, sex, and for past and present smoking.

Table 5 presents all-cause and cause-specific mortality comparing vegetarians to non-vegetarians. In all three cohorts, vegetarians experienced a 10% to 20% decreased in all-cause mortality. Similarly, vegetarians had 26% to 68% lower risks of mortality from ischemic heart disease, cardiovascular disease, and cerebrovascular disease. Vegetarians experienced a 48% risk reduction in mortality from breast cancer, and modest risks reduction from other-cause total mortality. No significant risks reduction in cause-specific mortality were found among vegetarians, particularly for cancers of the stomach, colorectal, lung, and prostate.

Table 6 presents cardiometabolic-related factors comparing vegan and lacto-ovo-vegetarian Adventists to the non-vegetarian counterparts. Lacto-ovo-vegetarians and vegans had respectively, 3 and 5 points lower BMI than non-vegetarians. For hypertension, lacto-ovo-vegetarians experienced 55% lower risks; whereas, vegans had 75% risks reduction when compared to non-vegetarians. Similarly, lacto-ovo-vegetarian and vegan were associated with lower risks of type-2 diabetes. The risks reduction of diabetes for lacto-ovo-vegetarians varied between 38% and 61%; and 47% to 78% for vegans. When stratified by ethnicity, the relative risks reduction for diabetes was greater in blacks than non-blacks when compared with their non-vegetarian counterparts. For all cardiometabolic-related outcomes, vegans had lower risk than lacto-ovo-vegetarians compared to non-vegetarians.

Table 7 presents all-cancer and site-specific cancers for vegan and lacto-ovo-vegetarian Adventists compared to non-vegetarians. Overall, vegans experienced modest risks reduction (14%) for all-cancer, but not significant for lacto-ovo-vegetarians. When stratified by gender, no significant differences were observed in the hazard ratio for all-cancer in vegans. Conversely, lacto-ovo-vegetarians conferred 24% risks reduction for cancer of the gastrointestinal tract, but not significant for vegans. Vegans experienced 73% higher risk for urinary tract cancer compared to non-vegetarians. Neither vegans nor lacto-ovo-vegetarians had significant risks reduction for cancer of the respiratory tract.

**Table 5:** All-cause and cause-specific mortality among vegetarian and non-vegetarian Adventists.

Cause-Specific Mortality	Person at-Risk	No. of Events	Parameter Estimates	Non-Vegetarian	Vegetarian	Cohort(s) & References	
				SMR, RR, or HR (95% CI)			
**All-cause** ^[a]^^,^*	24,538	1635	SMR	1 [*referent*]	0.83 (0.76, 0.92)	AMS	[25]
**All-cause** ^[a]^^,^*	28,952	3564	SMR	1 [*referent*]	0.80 (0.74, 0.87)	AHS-1	
**All-cause** ^[b]^^,^^‡^	73,308	2560	HR	1 [*referent*]	0.88 (0.80, 0.97)	AHS-2	[19]
*Males* ^[b]^^,^^‡^	25,105	1031	HR	1 [*referent*]	0.82 (0.72, 0.94)		
*Females* ^[b]^	48,203	1529	HR	1 [*referent*]	0.93 (0.82, 1.05)		
**Ischemic Heart Disease** ^[a]^^,^*	24,538	598	SMR	1 [*referent*]	0.74 (0.63, 0.88)	AMS	[25]
*Males* ^[c]^^,^^♠^	8195	33	RR	1 [*referent*]	0.32 (0.13, 0.89)	AMS	[26]
*Females* ^[d]^	14,888	211	RR	1 [*referent*]	0.83 (0.37, 2.63)		
**Ischemic Heart Disease** ^[a]^^,^*	28,952	921	SMR	1 [*referent*]	0.62 (0.53, 0.73)	AHS-1	[25]
**Ischemic heart disease** ^[b]^	73,308	372	HR	1 [*referent*]	0.81 (0.64, 1.02)	AHS-2	[19]
**Cardiovascular Disease** ^[b]^	73,308	987	HR	1 [*referent*]	0.87 (0.75, 1.01)		
*Males* ^[b]^^,^^‡^	25,105	390	HR	1 [*referent*]	0.71 (0.57, 0.90)		
*Females* ^[b]^	48,203	597	HR	1 [*referent*]	0.99 (0.83, 1.18)		
**Cerebrovascular Disease** ^[a]^^,^*	24,538	182	SMR	1 [*referent*]	0.65 (0.48, 0.87)	AMS	[25]
**Cerebrovascular Disease** ^[a]^	28,952	317	SMR	1 [*referent*]	0.93 (0.73, 1.19)	AHS-1	
**All-cancer** ^[b]^	73,308	706	HR	1 [*referent*]	0.92 (0.78, 1.08)	AHS-2	[19]
**Stomach** ^[a]^	24,538	30	SMR	1 [*referent*]	0.64 (0.30, 1.36)	AMS	[25]
**Stomach** ^[a]^	28,952	26	SMR	1 [*referent*]	1.58 (0.68, 3.70)	AHS-1	
**Colorectal** ^[a]^	24,538	41	SMR	1 [*referent*]	1.37 (0.73, 2.56)	AMS	
**Colorectal** ^[a]^	28,952	104	SMR	1 [*referent*]	1.01 (0.66, 1.56)	AHS-1	
**Lung** ^[a]^	24,538	6	SMR	1 [*referent*]	0.59 (0.10, 3.28)	AMS	
**Lung** ^[a]^	28,952	96	SMR	1 [*referent*]	0.69 (0.37, 1.27)	AHS-1	
**Breast** ^[a]^	24,538	26	SMR	1 [*referent*]	0.65 (0.28, 1.52)	AMS	
**Breast** ^[a]^^,^*	28,952	64	SMR	1 [*referent*]	0.52 (0.27, 0.97)	AHS-1	
**Prostate** ^[a]^	24,538	15	SMR	1 [*referent*]	1.41 (0.49, 4.04)	AMS	
**Prostate** ^[a]^	28,952	66	SMR	1 [*referent*]	0.79 (0.44, 1.41)	AHS-1	
**Other-cause** ^[a]^	24,538	737	SMR	1 [*referent*]	0.96 (0.83, 1.11)	AMS	
**Other-cause** ^[a]^^,^*	28,952	1970	SMR	1 [*referent*]	0.88 (0.79, 0.97)	AHS-1	
**Other-cause** ^[b]^	73,308	867	HR	1 [*referent*]	0.85 (0.73-0.99)	AHS-2	[19]

Non-vegetarian is the referent group. RR = relative risk; SMR = standard mortality ratio; HR = hazard ratio*;* ^[a]^ Adjusted for age, sex, and smoking status; death rate ratio (SMR); * *p* < 0.05, statistically significant; ^[b]^ Adjusted by age (*i*.*e*., attained age as time variable), race (black, nonblack), smoking, exercise, personal income, educational level, marital status, alcohol, region, and sleep. Also adjusted by sex, menopause, and hormone therapy. Other-cause includes non-CVD and non-cancer; ^‡^ *p* < *0*.*05*; ^[c]^ Adjusted for age and current dietary habits, 1960 to 1965; for males ages 35–64; RR = SMR in non-vegetarians/SMR in vegetarians; ^♠^ *p* = *0*.*0084*; ^[d]^ Adjusted for age and current dietary habits, 1960 to 1965; for females ages 35–74.

**Table 6:** Cardiometabolic-related factors among vegan and lacto-ovo-vegetarian Adventists.

Cardio-Metabolic Factors	Person at-Risk	No. of Events	Parameter Estimates	Lacto-ovo-Vegetarian	Vegan	Cohort(s) & References
				Mean, RR, or OR (95% CI)		
**Body mass index** ^[a]^^,^*	89,224	-	Mean	25.5 (25.4, 25.5)	23.1 (23.0, 23.2)	AHS-2 [27]
**Body mass index** ^[c]^^,^^‡^	60,903	-	Mean ± SD	25.7 ± 5.1	23.6 ± 4.4	AHS-2 [16]
**Hypertension** ^[a]^^,^*	89,224	-	RR	0.45 (0.44, 0.47)	0.25 (0.22, 0.28)	AHS-2 [27]
**Hypertension** ^[b],†^	500	-	OR	0.86 (0.51, 1.45)	0.53 (0.25, 1.11)	AHS-2 [18]
**Diabetes** ^[a]^^,^*	89,224	-	RR	0.39 (0.36, 0.42)	0.22 (0.18, 0.28)	AHS-2 [27]
**Type-2 Diabetes** ^[c]^^,^^‡^	60,903	3394	OR	0.54 (0.49, 0.60)	0.51 (0.40, 0.66)	AHS-2 [16]
**Diabetes Mellitus** ^[d]^^,^^♠^	41,387	616	OR	0.62 (0.50, 0.76)	0.38 (0.24, 0.62)	AHS-2 [28]
*Non-blacks* ^[d]^^,^^♠^	34,215	-	OR	0.68 (0.54, 0.86)	0.43 (0.25, 0.74)	
*Blacks* ^[d]^^,^^♠^	7172	-	OR	0.47 (0.27, 0.83)	0.30 (0.11, 0.84)	

Non-vegetarian is the referent group. RR = relative risk; OR = odd ratio; ^[a]^ Adjusted for age, sex, and race. Body mass index (BMI) for non-vegetarians [*reference*] was 28.26 (28.22, 28.30); * *p* = 0.0001; ^[b]^ Adjusted for age, gender and BMI. Hypertension defined as average systolic blood pressure >139 mmHg or average diastolic blood pressure >89 mmHg or taking antihypertensive medications; ^†^ *p* = 0.0001; ^[c]^ Adjusted for age, sex, ethnicity, education, income, physical activity, television watching, sleep habits, alcohol use, and BMI. Body mass index (BMI) for non-vegetarians referent group was 28.8 ± 6.3; ^‡^ *p* = 0.0001; ^[d]^ Adjusted for age, gender, BMI, lifestyle, sleep habits, physical activity, alcohol use, ethnicity, income, and education. Type-1 diabetes were excluded; ^♠^ *p* < 0.05, statistically significant.

**Table 7:** All-cancer and site-specific cancers among vegan and lacto-ovo-vegetarian Adventists.

Cancer-Specific Sites	Person at-Risk	No. of Events	Parameter Estimates	Lacto-ovo-Vegetarian	Vegan	Cohort(s) & References
				HR (95% CI)		
**All-Cancer** ^[a]^^,^*	69,120	1068	HR	0.95 (0.86, 1.04)	0.86 (0.73, 1.00)	AHS-2 [20]
*Males* ^[b]^	24,446	457	HR	0.92 (0.80, 1.06)	0.81 (0.64, 1.02)	
*Females* ^[a]^	44,674	611	HR	0.96 (0.85, 1.08)	0.91 (0.75, 1.11)	
**Gastrointestinal** ^[c]^^,^^‡^	69,120	166	HR	0.76 (0.61, 0.94)	0.80 (0.55, 1.17)	
**Respiratory Tract** ^[c]^	69,120	54	HR	0.85 (0.61, 1.30)	0.59 (0.28, 1.23)	
**Urinary Tract** ^[c]^	69,120	80	HR	1.13 (0.79, 1.61)	1.73 (1.05, 2.84)	
**All-male cancer** ^[d]^	24,446	205	HR	0.95 (0.66, 1.25)	0.81 (0.57, 1.17)	
**All-female cancer** ^[c]^	44,674	284	HR	1.04 (0.87, 1.25)	0.71 (0.50, 1.01)	

All-female cancer includes female breast, vulva, vagina, cervix uteri, corpus uteri, endometrial, uterus, and ovary. Cancer of the gastrointestinal tract includes esophagus, stomach, small intestine, colon, liver and bile ducts, gallbladder, biliary tract, and pancreas. All-male cancer includes prostate, penis, and testis. Cancer of the respiratory tract and intra-thoracic organs include nasal cavity, middle ear, larynx, trachea, bronchus, lung, heart, mediastinum, and pleura. Cancer of the urinary tract includes renal pelvis, ureter, kidney, and bladder; Non-vegetarian is the referent group*.* HR = hazard ratio; ^[a]^ HR adjusted by race, family history of cancer, BMI, education, smoking, alcohol, age at menarche, pregnancies, breastfeeding, oral contraceptives, hormone replacement therapy, and menopause status; * *p* = 0.03; ^[b]^ HR adjusted by race, family history of cancer, BMI, education, smoking, and alcohol; ^[c]^ Multivariate HR model adjusted by race, family history of cancer, education, smoking, alcohol, age at menarche, pregnancies, breastfeeding, oral contraceptives, hormone replacement therapy, menopause status, and BMI; ^‡^ *p* = 0.001; ^[d]^ Multivariate HR model adjusted for age, family history of cancer, education, smoking, alcohol, and BMI.

Table 8 presents results for all-cause and cause-specific mortality comparing lacto-ovo-vegetarian and vegan Adventists to non-vegetarians. Lacto-ovo-vegetarians experienced a 9% risks reduction for all-cause total mortality. Vegan had 14% lower risks of all-cause mortality in males. Lacto-ovo-vegetarian and vegan males experienced a 23% and 42% risks reduction in CVD mortality, respectively. Vegan males also had a 55% risk reduction for ischemic heart disease. Overall, the risks reduction in all-cause and specific-cause mortality were greater in males than females.

**Table 8:** All-cause and cause-specific mortality among lacto-ovo-vegetarians and vegan Adventists.

Cause-Specific Mortality	Person at-Risk	No. of Events	Parameter Estimates	Lacto-ovo-Vegetarian	Vegan	Cohort(s) & References
				HR (95% CI)		
**All-cause mortality** ^[a],[b]^^,^^‡^	73,308	2560	HR	0.91 (0.82, 1.00)	0.85 (0.73, 1.01)	AHS-2 [19]
*Males* ^[a]^^,^*	25,105	1031	HR	0.86 (0.74, 1.01)	0.72 (0.56, 0.92)	
*Females* ^[c]^	48,203	1529	HR	0.94 (0.83, 1.07)	0.97 (0.78, 1.20)	
**All-Cancer** ^[a],[b]^	73,308	706	HR	0.90 (0.75, 1.09)	0.92 (0.68, 1.24)	
*Males* ^[a]^	25,105	273	HR	1.01 (0.75, 1.37)	0.81 (0.48, 1.36)	
*Females*	48,203	433	HR	0.85 (0.67, 1.09)	0.99 (0.69, 1.44)	
**Ischemic Heart Disease** ^[a],[b]^	73,308	372	HR	0.82 (0.62, 1.06)	0.90 (0.60, 1.33)	
*Males* ^[a]^^,^*	25,105	159	HR	0.76 (0.52, 1.12)	0.45 (0.21, 0.94)	
*Females* ^[c]^	48,203	203	HR	0.85 (0.59, 1.22)	1.39 (0.87, 2.24)	
**Cardiovascular Disease** ^[a], [b]^	73,308	987	HR	0.90 (0.76, 1.06)	0.91 (0.71, 1.16)	
*Males* ^[a]^^,^*	25,105	390	HR	0.77 (0.59, 0.99)	0.58 (0.38, 0.89)	
*Females* ^[c]^	48,203	597	HR	0.99 (0.81, 1.22)	1.18 (0.88, 1.60)	
**Other-cause** ^[a],[b]^^,^^‡^	73,308	867	HR	0.91 (0.77–1.07)	0.74 (0.56–0.99)	
*Males* ^[a]^	25,105	368	HR	0.89 (0.69–1.15)	0.81 (0.53–1.22)	
*Females* ^[c]^	48,203	499	HR	0.93 (0.75–1.17)	0.70 (0.47–1.05)	

Non-vegetarian is the referent group. HR = hazard ratio; ^[a]^ Adjusted by age (*i*.*e*., attained age as time variable), race, smoking, exercise, personal income, educational level, marital status, alcohol, region, and. Other-cause includes non-CVD and non-cancer; * *p* < 0.05;^[b]^ Also adjusted by sex, menopause, and hormone therapy; ^ ‡^ *p* < 0.05; ^[c]^ Also adjusted by menopause and hormone therapy.

## 4. Discussion

The published studies of North American Adventists paint a consistent picture across all three study cohorts, associating vegetarian diets with lower body mass index [21,22,29], coronary heart disease [19,30], hypertension [18,21], type 2 diabetes [15,21,23,28], and metabolic syndrome [29]. Similarly, vegetarians have lower risks for colon, gastrointestinal tract, prostate and overall cancer. Among males, vegetarian diets seem to be protective against all-cause mortality and CVD mortality. Results for some diseases such as breast cancer mortality are not always consistent within the earlier Adventist cohorts [26,31,32,33,34]. The lower risk among vegetarians of several high prevalent chronic diseases results in a greater life-expectancy compared to non-vegetarians. Although, Adventist males tend to live 7.3 years and females 4.4 years longer than their gender counterparts in the general Californian population [15,35], within the same cohort, vegetarians had more modest 1.5 to 2.4 years longer life-expectancy than non-vegetarian Adventists [35]. This suggests that other non-dietary factors of the Adventist lifestyles may also contribute to their longevity.

The other major vegetarian cohort is the European Prospective Investigation into Cancer and Nutrition (EPIC-Oxford) study where 65,429 participants were recruited from the United Kingdom, many of whom are vegetarians [36]. Similar to Adventist vegetarians, British vegetarians enjoy lower rate of cardiovascular disease and risk factors compared to their non-vegetarians British counterparts [37]. In the EPIC-Oxford cohort study [38,39,40], the total incidence of all cancers was lower among vegetarians than meat eaters; however, colorectal cancer rates were greater than the non-vegetarians [38]. Another study reported no difference in total mortality between British vegetarians compared with other health-conscious participants [41]. A pooled analysis has shown reductions only for IHD mortality [25]. A possible explanation for the discrepancy between British vegetarians and US Adventist vegetarians may be related to differences in diets other than the absence of meat [27]. Vegetarians and vegans from the Adventist cohorts consume more fiber and vitamin C than those of the EPIC-Oxford [29,36]. It has been previously noted that the lack of consistent findings from British vegetarians deserves further attention [27,42], since other non-dietary lifestyle factors may also differ between the two vegetarian cohorts.

Beyond meatless, there is a need to evaluate the health effects of different types of vegetarian diets. How does the health status of lacto-ovo-vegetarians and vegans differ from non-vegetarians? In the Adventist cohorts, both lacto-ovo-vegetarians and vegans have reduced risk for hypertension, type 2 diabetes, and obesity; however, vegans experienced greater risks reduction for those diseases. Similarly, both lacto-ovo-vegetarian males and vegan male have lower mortality from cardiovascular disease compared to non-vegetarians, but risk reduction is greater among vegans. In contrast, both lacto-ovo-vegetarian and vegan females do not seem to have a lower risk of mortality from cardiovascular diseases. Not so clear patterns are observed for cancer outcomes. While lacto-ovo-vegetarians have lower risk of cancer of the gastrointestinal tract, vegans experience a higher risk for cancer of the urinary tract. For other-cancer sites, the risk is slightly but not significantly lower for both lacto-ovo-vegetarians and vegans compared to non-vegetarians. Subsequent reports with longer follow-up time and more cancer cases will help clarify the role of specific vegetarian diets with cancer outcomes.

Several proposed mechanisms may explain the lower risks for some diseases exhibited by vegans compared to lacto-ovo-vegetarians. It is well established that obesity is a major risk factor for CVD [43,44,45,46] and cancer [47,48,49,50,51]. Significantly lower mean BMI observed in vegans may be an important factor for reducing risks from heart disease and CVD mortality [36]. Nutrient profiles of the vegetarian dietary patterns may explain cardiovascular risks reduction [29]. Dietary fibers, folate, antioxidants, and phytochemicals rich in fruits and vegetables [52,53], whole grains, soy, and nuts [54,55,56] are associated with low level of serum cholesterol [57], lower trans/saturated fat, lower incidence of diabetes [58], CVD [59], and specific cancers [60], and lower mortality incidence from stroke and IHD [61]. Other published studies of vegetarians also found that vegans are thinner [62], have lower LDL and total cholesterol [63] than lacto-ovo-vegetarians, among whites and African Americans [64]. Similarly, among Latin Americans, vegans have lower plasma lipids than lacto-ovo-vegetarians and omnivores [65]. Vinagre *et al*. [66] showed that vegan diets improved metabolic pathways of cholesterol remnant removal from circulation.

Vegans may have a greater challenge in meeting the nutritional adequacy for vitamin B_12_, protein, and calcium compared to lacto-ovo-vegetarians and meat-eaters. Thus, the potential adverse effects of vegan diets deserve consideration. In the EPIC-Oxford study [67], vegans had 30% higher fracture rates than meat-eaters. However, when adjusted for calcium intake, vegans no longer had higher fracture rate. Other studies found similar results [68]. In a review, Dunham *et al*. reported higher rates of vitamin B_12_ deficiency among vegans compared with other vegetarians [69]. Vitamin B_12_ deficiency may increase CVD risk factors [70], and is associated for a wide range of neurological disorders [71]. In addition, for those following a vegan diet, optimizing intakes of *n*-3 fatty acids is highly recommended [72,73,74]. Nevertheless, vegans can avoid nutritional inadequacy with appropriate food choices [4,7,72].

### Strengths and Limitations

The strength of this review is its focus on only prospective studies of vegetarian Adventists. The Adventist population places high emphasis on health and exhibits diverse dietary habits [14], with relatively low percentage of alcohol consumption and cigarette smoking. Thus, this reduces possible confounding due to non-dietary factors, and results in greater statistical power. In addition, the consistent and refined operational definitions of dietary patterns offer an advantage in measuring the relationship between vegetarian diets and disease/mortality outcomes.

Since this review is limited to the Adventist cohorts, the findings may not be fully generalizable to other vegetarians with different lifestyles. The studies in our review did not directly compare vegans with lacto-ovo-vegetarians, thus it may not provide adequate interpretation of the magnitude of differences in effect size between these two dietary patterns. The observed health benefits of vegetarians may be partially related to other lifestyle factors due to residual confounding. Dietary patterns may change over time, which has not been accounted for in the analysis. These studies, like any others using the food frequency questionnaires, include unavoidable measurement errors in the assessment of food consumptions. However, in the Adventist population, we have reported good validity for the foods used in defining the different types of vegetarian diets [75]. Non-vegetarian Adventists, used as a reference in all reported comparisons, consume much less meat than the general population [35,76]. Thus, the relatively low intake of meat by this reference group in this cohort may result in smaller observed effects. Relative small sample size within the vegan groups may limit our conclusions.

The research on the health effects of vegan diets and chronic diseases have mostly derived from observational studies of Adventist and Oxford vegetarians cohorts. These studies have mainly focused on cardiovascular disease and cancer outcomes. Further research is needed in the prospective investigation of obesity, diabetes, cognition, and other neurological disorders. Given the consistency of results from prospective epidemiological studies on vegetarian diets and cardiovascular disease, large randomized dietary intervention trials on vegan and lacto-ovo-vegetarian patterns are warranted to duplicate the findings and further investigate the health effects of these diets.

## 5. Conclusions

In summary, vegetarians have consistently shown to have lower risks for cardiometabolic outcomes and some cancers across all three prospective cohorts of Adventists. Beyond meatless diets, further avoidance of eggs and dairy products may offer a mild additional benefit. Compared to lacto-ovo-vegetarian diets, vegan diets seem to provide some added protection against obesity, hypertension, type-2 diabetes; and cardiovascular mortality. In general, the protective effects of vegetarian diets are stronger in men than in women. At present, there are limited prospective data on vegetarian dietary patterns and body weight change, obesity and neurological disorders. Large dietary intervention trials on the effects of vegetarian diets on obesity, diabetes, and cardiovascular outcomes are warranted to make meaningful recommendations for nutritional planning, assessment, and counseling.
